# The association between the body roundness index and the risk of colorectal cancer: a cross-sectional study

**DOI:** 10.1186/s12944-023-01814-2

**Published:** 2023-04-18

**Authors:** Wenxing Gao, Lujia Jin, Dingchang Li, Yue Zhang, Wen Zhao, Yingjie Zhao, Jingwang Gao, Lin Zhou, Peng Chen, Guanglong Dong

**Affiliations:** 1 Department of General Surgery, the First Clinical Medical Center of Chinese PLA General Hospital, No. 28 Fuxing Road, Beijing, 100853 China; 2 Medical School of Chinese PLA, Beijing, 100853 China; 3 School of Medicine, Nankai University, Tianjin, 300071 China; 4 Unit 69250 of Chinese PLA, Xinjiang, 830000 China

**Keywords:** Body roundness index, Abdominal obesity, Visceral fat, Colorectal cancer, Anthropometric index

## Abstract

**Background:**

Colorectal cancer (CRC), has a link between obesity, especially visceral fat. The body roundness index (BRI) can more accurately assess body fat and visceral fat levels. It is, however, unknown whether BRI is associated with CRC risk.

**Methods:**

53,766 participants were enrolled from the National Health and Nutrition Examination Survey (NHANES). Analysing the corelation between BRI and CRC risk was performed using logistic regression. Stratified analyses revealed the association based on the population type. Receiver operating characteristic curve (ROC) was performed for predicting CRC risk using different anthropometric indices.

**Results:**

The risk of CRC mounting apparently with elevated BRI for participants with CRC compared to normal participants (*P*-trend < 0.001). The association persisted even after adjusting for all covariates (*P*-trend = 0.017). In stratified analyses, CRC risk increased with increasing BRI, especially among those who were inactive (OR (95% CI): Q3 3.761 (2.139, 6.610), *P* < 0.05, Q4 5.972 (3.347, 8.470), *P* < 0.01), overweight (OR (95% CI): Q3 2.573 (1.012, 7.431), *P* < 0.05, Q4 3.318 (1.221, 9.020), *P* < 0.05) or obese (OR (95% CI): Q3 3.889 (1.829, 8.266), *P* < 0.001, Q4 4.920 (2.349, 10.308), *P* < 0.001). ROC curve showed that BRI had a better ability in forecasting the risk of CRC than other anthropometric indices such as body weight etc. (all *P* < 0.05).

**Conclusions:**

CRC risk and BRI have a positive and significant relationship, particularly in inactive participants with BMI ≥ 25 kg/m^2^. It is hoped that these results will raise awareness of the importance of reducing visceral fat deposition.

## Introduction

Colorectal cancer (CRC), whose prevalence ranked third and mortality ranked second among all cancers [1]. Many countries, including China, are experiencing a rise in CRC incidence, and new cases are occurring at an increasingly young age [2, 3]. The occurrence and development of CRC is a long-term, multistage process, and the environment, dietary habits, lifestyle, and genetics are all influential factors in the development of CRC [4]. This means that early detection and exclusion of CRC risk factors and early preventive colonoscopy can effectively reduce the incidence of CRC.

A significant risk factor for CRC is being obese, particularly abdominal obesity (visceral fat). There is a higher risk of CRC in obese individuals [5]. Excess adipose tissue, especially visceral fat in obese individuals, induces several pathophysiological processes associated with CRC, such as insulin resistance, chronic tissue inflammation, and oxidative stress [6]. Adipose tissue also secretes several cytokines and adipokines that affect intestinal immune system homeostasis, directly contributing to CRC progression and development [7, 8]. Computed tomography (CT) and magnetic resonance imaging (MRI) are effective for measuring accurate visceral fat, but the time consumption, high cost, and limited accessibility limit their application in everyday life. Bioelectrical impedance analysis is an easy-to-use, cost-effective, non-invasive technique to measure the body adiposity content [9]. Anthropometric indices, since they are non-invasive, cheap, and convenient [10], can be considered for population health screening and early risk finding. Therefore, anthropometric indices are commonly used to assess body fat in most studies.

Body roundness index (BRI) is a tool for measuring body fat and visceral fat, first introduced in 2013 [11]. As a result of its ability to reflect height-related body roundness (abdominal obesity) and provide an estimate of %body fat and %visceral fat that is more accurate, the BRI may be more useful than other anthropometric indices [11]. There is evidence that BRI is closely related to many human diseases, cardiovascular diseases [12–14], diabetes [15], and metabolic syndrome [16], for example. However, BRI and cancer have been little studied. There have only been a few studies linking BRI to liver and endometrial cancers [17, 18].

Based on 53,766 individuals’ data from the National Health and Nutrition Examination Survey (NHANES), the purpose of this study is to examine the correlation between BRI and CRC risk and provide ideas and strategies for early detection of CRC.

## Materials and methods

### Study participants and research design

**Fig. 1:**
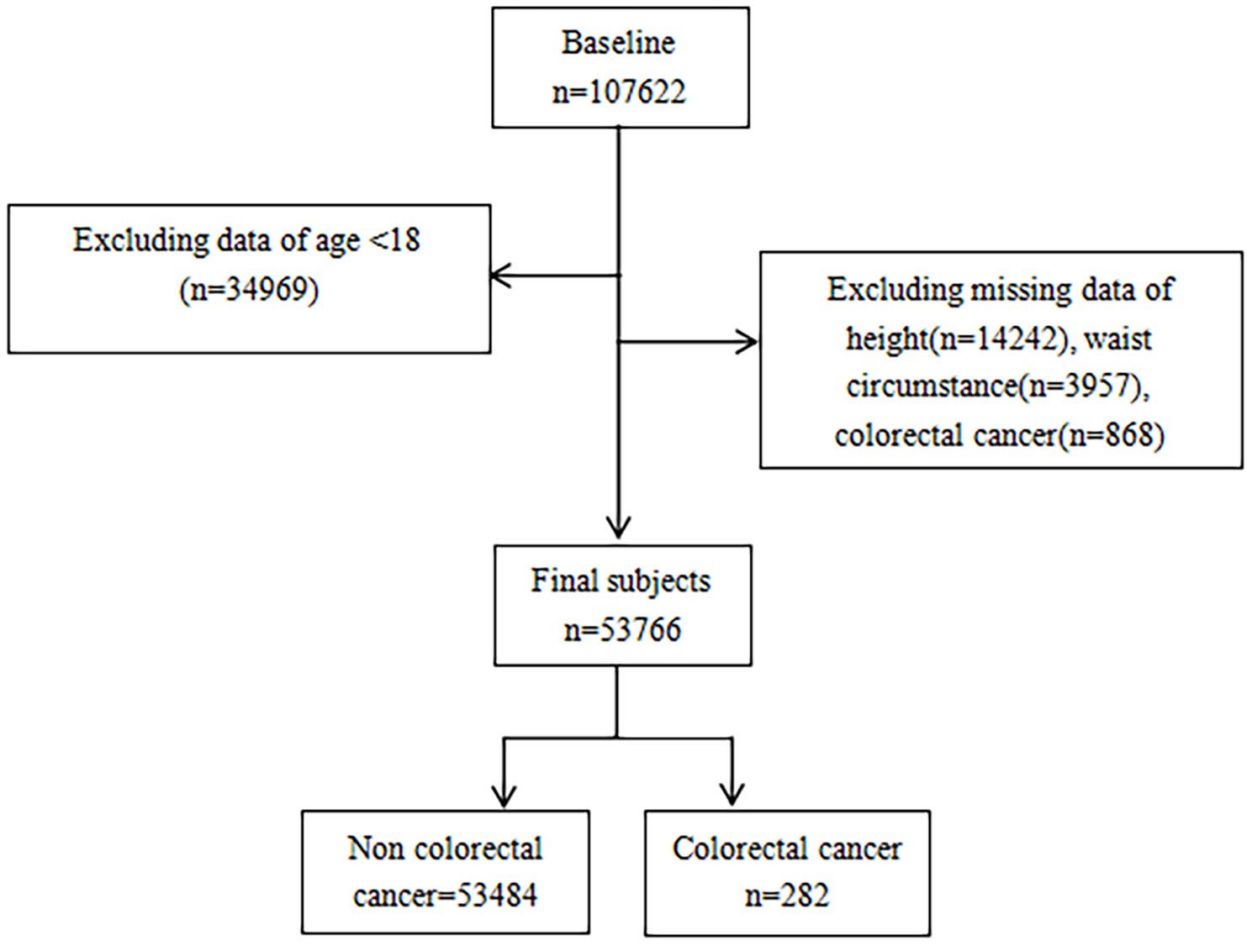
Flow chart of the study

An annual study of the health of the American population, the NHANES is conducted in the United States, and all data are directly available through an online website. All participants provided written informed consent. For this analysis, the inclusion criteria were all participants from 1999 to 2020, and participants whose age < 18 years old and lacking important clinical data were excluded. 53,766 participants were ultimately enrolled (Fig. 1).

### Data collection and measurements

Data were obtained from the NHANES database website. The database contains demographic data, including race, gender, age, etc.; dietary data; anthropometric data containing waist circumference (WC), weight, body mass index (BMI), etc.; laboratory data including albumin, glycated haemoglobin (HbA1c), etc.; and questionnaire data, including drinking habits, smoking habits, medical conditions, etc. A team of trained researchers collected basic information about participants. Regularly trained health technicians gathered blood and urine samples and anthropometric measurements at the mobile examination centre (MEC) and sent them to designated testing centres for uniform testing.

### Definition of variables

The presence or absence of CRC was collected by self-reports. The NHANES questionnaire contained information on age, gender, race, smoking, alcohol consumption, and exercise. Participants were classified according to their alcohol intake habits in the past 12 months: non-drinkers, occasional drinkers, and regular drinkers. There were three categories of smokers, namely non-smokers, occasional smokers, and regular smokers. We defined exercisers as those who engaged in moderate-intensity exercise, fitness, or recreational activities during the week. Taking weight (kg) and height (m) squared and dividing them together gives BMI. WC (cm) divided by height (cm) results in waist-to-height ratio (WHtR). BRI was computed by 364.2-365.5*(1-[WC(m)/2π]^2^/[0.5*height(m)]^2^) ^½^ [11].

### Statistical analysis

Univariate analysis and comparison of individual baseline characteristics between participants were performed first. Percentages (%) were conducted to express categorical variables, and differences were analysed by chi-square tests. For continuous variables, Kolmogorov-Smirnov test was performed, and reported values were represented by mean and standard deviation. The skewness distribution variable was expressed as median and quartile spacing, comparing groups using Mann-Whitney U tests. An analysis of multivariable logistic regression models was conducted, for exploring the relationship between BRI quartiles with cancer risk. Throughout the analysis, the lowest quartile was regarded as a reference. The ultimate model was adjusted for race, gender, age, fasting blood glucose (FBG), exercise, total cholesterol (TC), smoking habits, drinking habits, triglycerides (TG), albumin, and low-density lipoprotein cholesterol (LDL-C). Odds ratios (OR) and 95% confidence intervals (CI) conducted to present the final results. Stratified analysis was conducted for gender, exercise, and BMI, to further explore the correlation between BRI quartiles with CRC risk consequently. Finally, after adjustment for covariates, receiver operating characteristic (ROC) curve was analysed. Moreover, the area under the receiver operating characteristic curve (AUC) of BRI with weight, BMI, WC, and WHtR were compared. IBM SPSS Statistics Version 26.0 and MedCalc Statistical Software Version 19.7.4 were used to analyse the data. There was statistical significance if the *P* value was less than 0.05 on a two-sided basis.

## Results

### Characteristics of study participants at the baseline

At the baseline level of the study, 53,766 individuals were enrolled, 26,106 males and 27,760 females. Demographic and clinical data about the study participants were displayed in Table 1, which were based on the diagnosis of CRC. Different from the non-CRC group, the CRC group was generally older, exercised less, and had significantly higher levels of TG (1.61 vs. 1.41), LDL-C (2.90 vs. 2.85), and FBG (6.22 vs. 5.61) (all *P* ≤ 0.001). BMI (28.65 vs. 27.80), WC (101.95 vs. 97.20), weight (77.65 vs. 67.70), WHtR (0.57 vs.0.53) and BRI (5.67 vs. 4.13), all of these are a higher level for the CRC group (all *P* < 0.001).

**Table 1:** Baseline characteristics

Variables	Total	Non colorectal cancer	Colorectal cancer	*P* value
Number	53,766	53,484	282	
Age, years	47.00 (31.00)	47.00 (31.00)	72.00 (19.00)	< 0.001
Men, n (%)	26,106 (48.6)	25,960 (48.5)	146 (51.8)	0.001
Race, n (%)				< 0.001
Mexican American	9430 (17.5)	9399 (17.6)	31 (11.0)	
Other Hispanic	4553 (8.5)	4537 (8.5)	16 (5.7)	
Non-Hispanic White	22,996 (42.8)	22,840 (42.7)	156 (55.3)	
Non-Hispanic Black	11,713 (21.8)	11,651 (21.8)	62 (22.0)	
Other	5074 (9.4)	5057 (9.5)	17 (6.0)	
Smoking habits, n (%)				< 0.001
No	12,699(53.8)	12,578 (53.7)	121(75.6)	
Occasional	2067 (8.8)	2061 (8.8)	6 (3.8)	
Regular	8822 (37.4)	8789 (37.5)	33 (20.6)	
Drinking habits, n (%)				< 0.001
No	9289 (22.0)	9189 (21.9)	100 (41.7)	
Occasional	17,874 (42.4)	17,801 (42.4)	73 (30.4)	
Regular	15,036 (35.6)	14,969 (35.7)	67 (27.9)	
Exercise, n (%)				< 0.001
Yes	22,535 (43.7)	22,426 (43.8)	109 (38.9)	
No	28,984 (56.3)	28,813 (56.2)	171 (61.1)	
TC, mmol/L	4.89 (1.50)	4.89 (1.50)	4.81 (1.81)	< 0.001
TG, mmol/L	1.41 (1.84)	1.41 (1.84)	1.61 (1.875)	0.001
HDL-C, mmol/L	1.29 (0.51)	1.31 (0.51)	1.29 (0.54)	< 0.001
LDL-C, mmol/L	2.85 (1.21)	2.85 (1.21)	2.90 (1.32)	< 0.001
FBG, mmol/L	5.65 (1.39)	5.61 (1.34)	6.22 (2.17)	< 0.001
HbA1c, (%)	5.50 (0.60)	5.50 (0.60)	5.70 (0.90)	0.001
Albumin, g/dL	41.00 (17.00)	41.00 (16.00)	40.00 (38.60)	< 0.001
BMI, kg/m^2^	27.80 (8.15)	27.80 (8.15)	28.65 (7.93)	< 0.001
WC, cm	97.20 (21.30)	97.20 (21.38)	101.95 (22.45)	< 0.001
Height, cm	166.80 (14.60)	166.80 (14.60)	166.65 (14.90)	0.004
WHtR	0.53(0.08)	0.53(0.07)	0.57(0.08)	< 0.001
BRI	4.14 (2.92)	4.13 (2.89)	5.67 (2.77)	< 0.001

Notes: Data are expressed as median (IQR) for skewed variables and percentage (%) for categorical variables

Abbreviations: BMI: body mass index; BRI: body roundness index; FBG: fasting blood glucose; HbA1c: glycosylated haemoglobin; HDL-C: high-density lipoprotein cholesterol; IQR: interquartile range; LDL-C: low-density lipoprotein cholesterol; TC: total cholesterol; TG: triglyceride; WC: waist circumstance; WHtR: waist-to-height ratio

### Associations between BRI and the risk of CRC

Followed by a correlation analysis between BRI and CRC risk using progressively adjusted multivariate regression, the first quartile interval of BRI as a control (Table 2). In general, BRI was positively correlated with CRC risk in Model 1, and CRC risk increased with increasing BRI (*P*-trend < 0.001). In Model 2, BRI was statistically significant only when it was at high values (OR (95% CI): Q3 1.757 (1.153, 2.678), Q4 1.891 (1.247, 2.867), *P*-trend < 0.001). Then, all confounders were adjusted for Model 3, BRI remained correlated with the risk of CRC (OR (95% CI): Q3 2.663 (1.002, 7.076), Q4 2.771 (1.025, 7.171), *P*-trend = 0.017). This suggests an association between BRI and CRC risk.

**Table 2:** Association between BRI quartiles and the risk of CRC in participants

Variables	BRI Quartiles				
	Q1	Q2	Q3	Q4	*P* for trend
Model 1					
OR (95% CI)	1	1.669(1.061, 2.626)	3.250(2.157, 4.898)	3.519(2.344, 5.284)	
*P* value		0.027	< 0.001	< 0.001	< 0.001
Model 2					
OR (95% CI)	1	1.065(0.673, 1.647)	1.757(1.153, 2.678)	1.891(1.247, 2.867)	
*P* value		0.789	0.003	0.009	< 0.001
Model 3					
OR (95% CI)	1	1.881(0.681, 5.195)	2.663(1.002, 7.076)	2.771(1.025, 7.171)	
*P* value		0.223	0.045	0.048	0.017

Model 1: unadjusted; Model 2: adjusted for age, race, sex; Model 3: further adjusted for exercise, drinking habits, smoking habits, albumin, FBG, TG, TC and LDL-C based on Model 2

Abbreviations: BMI: body mass index; BRI: body roundness index; CI, confidence interval; FBG: fasting blood glucose; LDL-C: low-density lipoprotein cholesterol; OR, odds ratio; TC: total cholesterol; TG: triglyceride

### Stratification analysis based on gender, exercise, and BMI

**Fig. 2:**
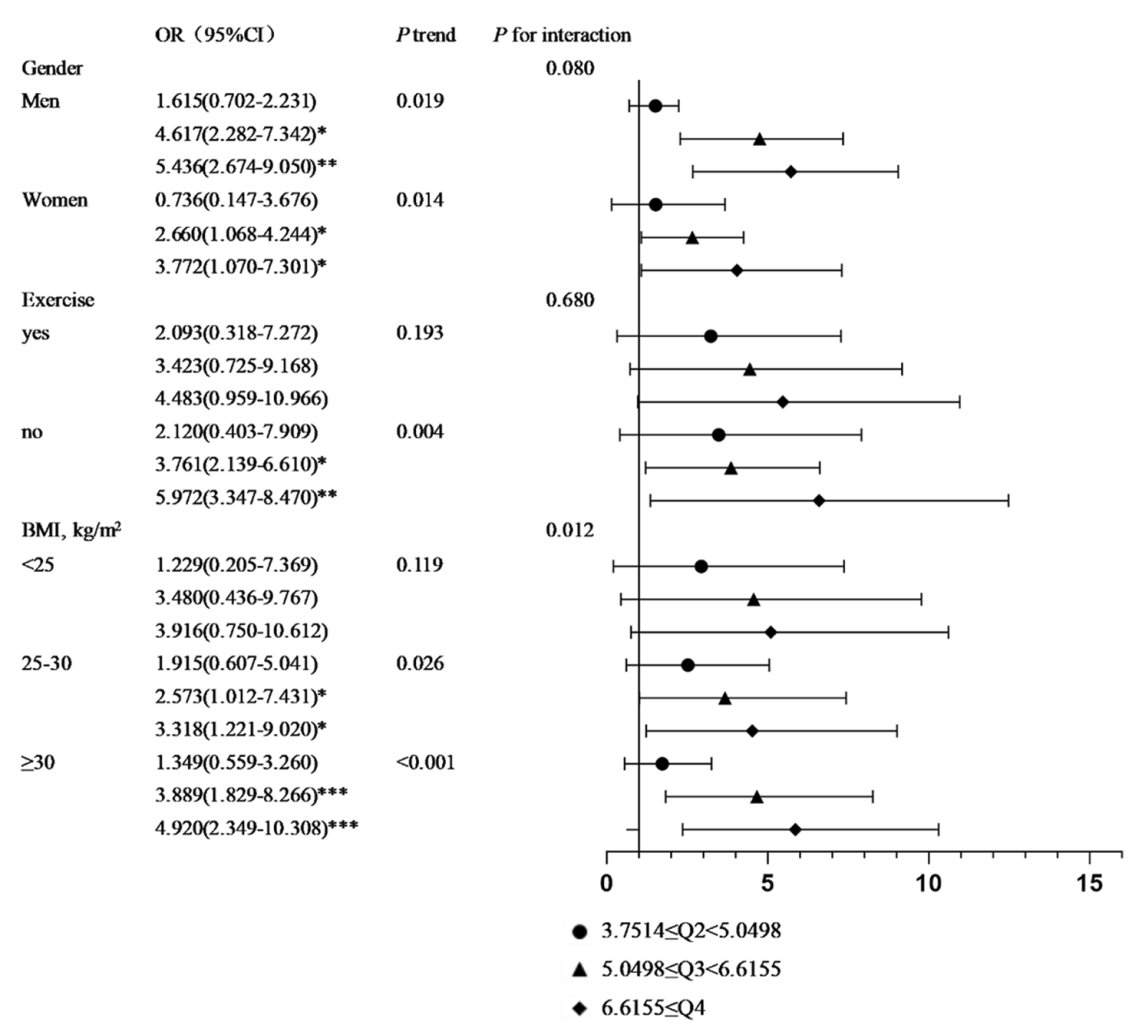
Association between BRI and CRC risk in different participants. Notes: *, *P* value < 0.05; **, *P* value < 0.01; ***, *P* value < 0.001

By using a stratified analysis in conjunction with Model 3, the stability of the correlation between BRI and CRC risk was further confirmed in different populations (Fig. 2). As a result of stratification by gender, BRI was significantly correlated with CRC risk in the third and fourth quartile intervals in men (OR (95% CI): Q3 4.617 (2.282, 7.342), Q4 5.436 (2.674, 9.050), *P*-trend = 0.019). The same was true among women (OR (95% CI): Q3 2.660 (1.068, 4.244), Q4 3.772 (1.070, 7.301), *P*-trend = 0.014). In stratified analyses by whether or not they exercised, a significant mounting in association between CRC with BRI in those who did not exercise (OR (95% CI): Q3 3.761 (2.139, 6.610), Q4 5.972 (3.347, 8.470), *P*-trend = 0.004)). Stratified by BMI (< 25 kg/m^2^; 25–30 kg/m^2^; ≥30 kg/m^2^), the most common anthropometric index in daily life, the risk of CRC elevated with mounting BRI, especially in participants with a BMI of 25–30 kg/m^2^ (OR (95% CI): Q3 2.573 (1.012, 7.431), Q4 3.318 (1.221, 9.020), *P*-trend = 0.026) and BMI greater than 30 kg/m^2^ (OR (95% CI): Q3 3.889 (1.829, 8.266), Q4 4.920 (2.349, 10.308), *P*-trend < 0.001).

### BRI can better predict CRC risk

**Fig. 3:**
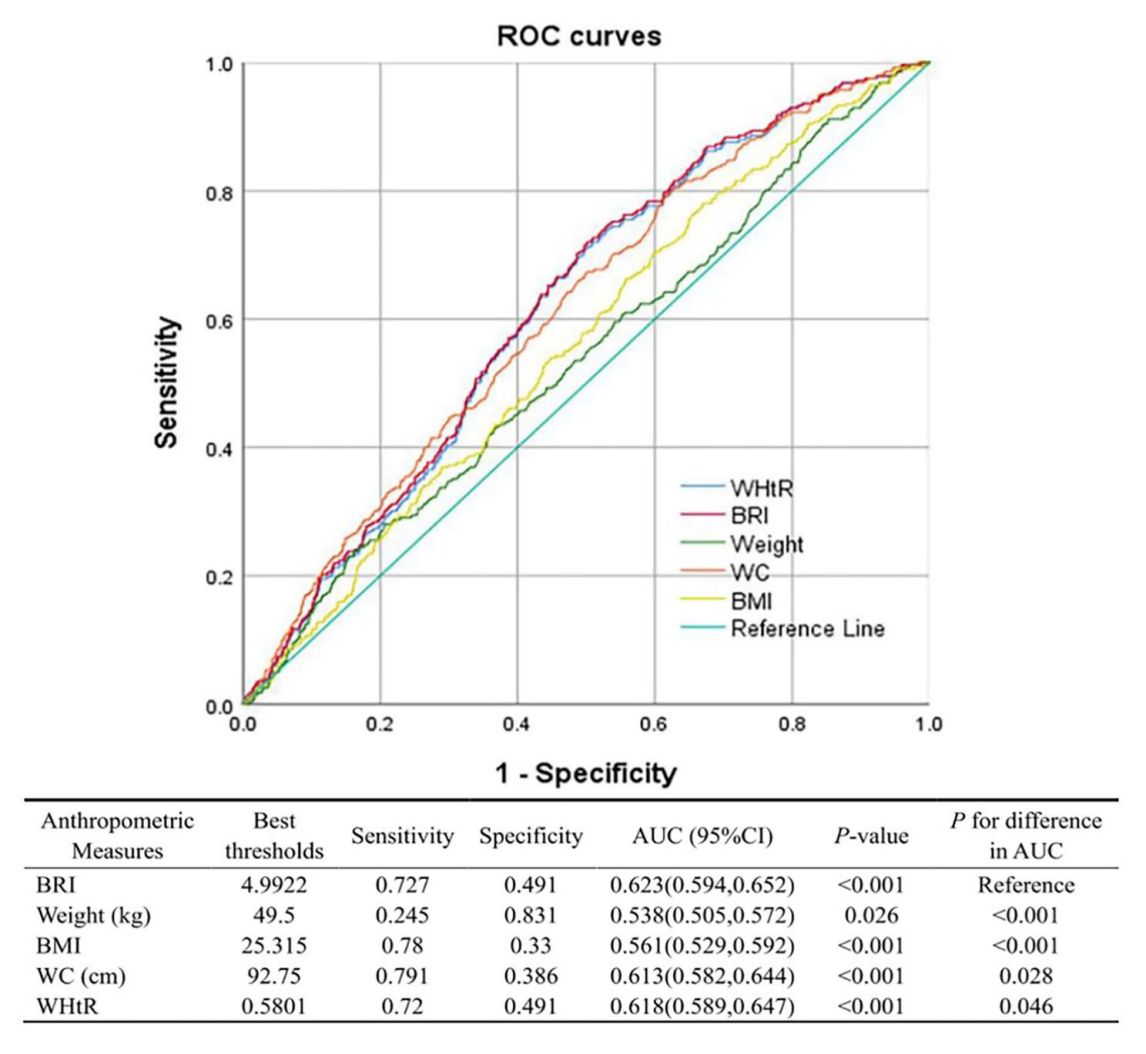
ROC curves of different anthropometric indices for the prediction of CRC risk

BMI and weight are the most extensively used anthropometric indices, while commonly used indices reflecting central obesity are WC and WHtR. Therefore, AUC was calculated to contrast the accuracy of BRI and different anthropometric indices in forecasting CRC risk (Fig. 3). In our study, BRI performed better than all five anthropometric indices (AUC (95% CI): 0.623 (0.594–0.652), all *P* < 0.05), The optimal cut-off value for BRI was 4.9922.

## Discussion

The BRI level was link to elevated risk for CRC in this study. The correlation existed persistently after full adjustment for confounders such as age, gender, exercise, smoking, alcohol consumption, albumin, FBG, TC, TG, and LDL-C, suggesting that CRC might be influenced by BRI. In addition, individuals with a higher BRI showed a higher risk of CRC, according to the stratified analysis, particularly among those who were inactive, overweight, and obese. Next, sensitivity analysis revealed that BRI had a better ability to predict CRC risk than weight, BMI, WC, and WHtR. BRI has rarely been linked to cancer, and this relationship is examined specifically for the first time in this study. Detecting CRC at an early stage is crucial to treating it. Exercise and weight control to reduce fat, especially visceral fat, can help prevent the disease and reduce its risk, benefiting the population even more.

Obesity, as a chronic inflammatory condition, is closely related to cardiovascular diseases, diabetes, cancer, and metabolic syndrome in humans [19–21]. Increased abnormal adipose tissue, especially visceral adipose tissue, can cause an imbalance in body homeostasis by secreting inflammatory factors and lipid factors, leading to pathological processes such as oxidative stress, insulin resistance, and altered cell proliferation [22–25]. The traditional anthropometric indices used to measure the degree of obesity are weight, BMI, WC, WHtR, etc. Weight and BMI are the most widely used but have limitations that cannot assess the specific distribution of body fat. WC and WHtR are mostly used to assess central obesity but cannot distinguish visceral fat from subcutaneous fat, which is more harmful. Therefore, new anthropometric indices have been continuously developed and applied. The standard for measuring visceral fat distribution is MRI and CT. However, they are time-consuming, expensive, and very inconvenient to use daily. BRI, as a promising index which reflects height-related body roundness and can be devoted to estimate visceral fat levels more accurately than traditional anthropometric indices. BRI has been reported to be an effective predictor of diabetes (including prediabetes), cardiometabolic diseases, and metabolic syndrome [13–16, 26]. Increased BRI is significantly related to cardiovascular disease outcomes and even all-cause mortality [12]. In addition, studies predicting cancer risk by anthropometric indices have also been reported [27–29]. In conclusion, as a marker reflecting body fat and visceral fat levels, BRI is expected to be an emerging health screening indicator and disease risk predictor.

Currently, cancer-related deaths from CRC rank second worldwide, and epidemiological studies prove that obesity links with cancer [30–32]. A total of 1.347 million new cases of CRC worldwide in 2012 were attributed to obesity in 5.8% of men and 7.0% of women [33]. Similarly, a study has inspected a summary of more than 1,000 epidemiological research programs and discovered that subjects with a BMI > 25 kg/m^2^ had a 1.2-1.5-fold elevated risk of CRC and that those with a BMI > 30 kg/m^2^ had a 1.5-1.8-fold elevated risk of CRC [30]. In addition, it was also shown that obesity was strongly connected with early-onset CRC with an approximate relative risk (RR) of 1.54 (1.01–2.35) [34]. Being overweight and obese in childhood and adolescence can lead to an increased incidence of early CRC [35, 36]. CRC usually develops from adenoma or polyp carcinoma, and a recently published meta-analysis indicated that excessive BMI was an isolated risk of adenomas and polyps and reported that the risk for colorectal adenoma (CRA) consistently increased by 42-44% in the overweight population and obesity population using controls with BMI < 25 kg/m^2^ [37]. Therefore, early and regular CRC screening is necessary for the obese population. Despite this, studies have shown that CRC screening compliance is significantly lower in obese populations than in normal populations. Research shows that obesity affects the likelihood of CRC screening discontinuation [38]. A Dutch survey also found that obesity was related to an obvious reduction in screening for CRC (OR: 0.8, 0.66–0.97) [39, 40]. This is unclear for why obese people have a lower adherence to CRC screening recommendations. Therefore, anthropometric indices to predict the risk of disease are a convenient, simple, quick, and more acceptable means.

BRI is investigated in the study as a potential risk factor for CRC and additional evidence is provided to validate the corelation between visceral fat with CRC risk as evaluated by BRI. BRI has better predictive ability than traditional anthropometric indices. Compared with screening methods such as colonoscopy, MRI, and CT, it saves time and effort. Simultaneously, this study should cause broader concern in clinical practice about visceral adipose distribution instead of just concentrating on reducing disease risk by losing weight. This study encourages the experimentation with BRI as an indicator to assess CRC risk in daily clinical practice.

## Comparisons with other studies and what does the current work add to the existing knowledge

The harm of visceral fat has attracted increasing attention, and in line with this study, most previous research on the visceral fat and CRC have confirmed the apparent correlation between them. Research has shown that CRC risk increases apparently with mounting WC and WHtR and shows a dose-dependent response [41, 42]. Compared with BMI, WC can better predict the occurrence of advanced CRC and more accurately reflect visceral fat levels with stronger carcinogenic effects [43]. Additionally, Dong et al. revealed that higher WHtR was related to elevated CRC risk (RR 95% CI: 1.39 (1.25, 1.53)) [44]. A study by Abal L., et al. also found that CRC was positively correlated with WC as well as between CRC and CRA [45]. However, some studies have also found no association between visceral fat assessed by WC and CRC [46], possibly as the result of an insufficient samples and single sample source. A link between visceral fat index, BRI, and CRC is explored in this study, filling the gap between BRI and CRC research currently available. Simultaneously, in this study, BRI showed superior predictive power to anthropometric measures including weight, BMI, WC, and WHtR. A significant link was observed between BRI and CRC risk even after correcting for confounding factors, indicating that BRI has the potential to be a predictor of CRC risk.

## Study strengths and limitations

Until now, no study has explored the relationship between BRI with CRC risk in the general population, and the large sample size is another advantage of this study. However, a few limitations also exist. Firstly, because the cross-sectional nature limited its ability to elucidate cause and effect, it is not sufficient to suggest that BRI are causally related to CRC risks. Secondly, the sample data were obtained from the NHANES database in the United States, and the regional and ethnic differences that exist limit the generalization and applicability of the findings to some extent. Thirdly, this study only analysed whether BRI correlates with overall CRC risk and did not evaluate the corelation among BRI to the progress, treatment, and prognosis of CRC. Finally, because the classification of the presence or absence of colorectal cancer was based on self-report, some recall bias should be considered. Future more detailed and in-depth studies are needed to further validate the relationship in follow-up.

## Conclusion

This study indicates that increased BRI is significantly linked to CRC risk, especially in inactive, overweight, and obese individuals. Compared with traditional anthropometric indicators, for example body weight, BMI, WC, and WHtR, BRI is more powerful in predicting the risk of CRC, and it is more convenient and efficient than the measurement of visceral fat by MRI or CT. This study aims to raise public awareness of the corelation between visceral fat represented by BRI and cancer and to encourage people to actively reduce body weight through diet control, regular exercise and other methods, and especially to reduce visceral fat deposition.

## Data Availability

The data are publicly available on the NHANES website: https://www.cdc.gov/nchs/nhanes/Index.htm.

## List of abbreviations

AUC: area under the curve
BMI: body mass index
BRI: body roundness index
CIs: confidence intervals
CRA: colorectal adenoma
CRC: colorectal cancer
CT: computed tomography
FBG: fasting blood glucose
HbA1c: glycated haemoglobin
IQR: interquartile range
LDL-C: low-density lipoprotein cholesterol
MEC: mobile examination centre
MRI: magnetic resonance imaging
NHANES: the National Health and Nutrition Examination Survey
ORs: odds ratios
ROC: receiver operating characteristic
TG: triglycerides
TC: total cholesterol
WC: waist circumference
WHtR: waist-to-height ratio.

## Notes

The original online version of this article was revised: the authors identified an error in figure 1 caption and would like to remove the supplementary material.
