# Correction to: The association between the body roundness index and the risk of colorectal cancer: a cross-sectional study

**DOI:** 10.1186/s12944-023-01824-0

**Published:** 2023-05-08

**Authors:** Wenxing Gao, Lujia Jin, Dingchang Li, Yue Zhang, Wen Zhao, Yingjie Zhao, Jingwang Gao, Lin Zhou, Peng Chen, Guanglong Dong

**Affiliations:** 1 Department of General Surgery, the First Clinical Medical Center of Chinese PLA General Hospital, No. 28 Fuxing Road, Beijing, 100853 China; 2 Medical School of Chinese PLA, Beijing, 100853 China; 3 School of Medicine, Nankai University, Tianjin, 300071 China; 4 Unit 69250 of Chinese PLA, Xinjiang, 830000 China

**Correction: Lipids in Health and Disease 22, 53 (2023)**

10.1186/s12944-023-01814-2

Following publication of the original article [1], the authors identified an error in Fig. 1 caption and would like to remove the supplementary material.

The original article [1] has been updated.
